# Nurses Needed

**Published:** 1918-09

**Authors:** 


### Nurses Needed.

The Government is making calls for more nurses. Some of these will be sent to France after they receive their training and others will be kept in this country to render necessary services here. But whether those who are thus trained ever get in the service of the country or not, the training itself will fit them for the duties of life. They will be better mothers for knowing how to administer to the sick; they will make better wives; and they will be more useful citizens. Young women who are not already engaged in some service for which they are adapted and who are not now living up to their full obligations as useful citizens should seriously consider nursing under Government training and direction as an occupation that will greatly increase their usefulness. There should be no parasites in the country now; every person who is not an invalid should be busy.

